# A real-world pharmacovigilance study of FDA adverse event reporting system (FAERS) events for sunitinib

**DOI:** 10.3389/fphar.2024.1407709

**Published:** 2024-07-24

**Authors:** Xusheng Zhang, Xiuli Ren, Tianyu Zhu, Wanjin Zheng, Chengwu Shen, Cuicui Lu

**Affiliations:** ^1^ Department of Pharmacology, Shandong Provincial Hospital Affiliated to Shandong First Medical University, Jinan, China; ^2^ Cheeloo College of Medicine, Shandong University, Jinan, China; ^3^ Department of Pharmacology, Hospital for Skin Diseases, Shandong First Medical University, Jinan, China; ^4^ Department of Pharmacology, Shandong Provincial Institute of Dermatology and Venereology, Shandong Academy of Medical Sciences, Jinan, China

**Keywords:** sunitinib, FDA adverse event reporting system, disproportionality analyses, pharmacovigilance, adverse event

## Abstract

**Background:**

Sunitinib is approved for the treatment of metastatic renal cell carcinoma (mRCC), imatinib-resistant gastrointestinal stromal tumors (GIST), and advanced pancreatic neuroendocrine tumors (PNET). This study aims to investigate the safety profiles of sunitinib through data mining of the US Food and Drug Administration Adverse Event Reporting System (FAERS).

**Methods:**

The individual case safety reports (ICSRs) on sunitinib from 2006 Q1 to 2024 Q1 were collected from the ASCII data packages in the Food and Drug Administration Adverse Event Reporting System (FAERS). After standardizing the data, a variety of disproportionality analyses, including the reporting odds ratio (ROR), the proportional reporting ratio (PRR), the bayesian confidence propagation neural network (BCPNN), and the multi-item gamma Poisson shrinker (MGPS) were employed to identify the potential safety signals of sunitinib-associated AEs.

**Results:**

A total of 35,923 ICSRs of sunitinib as the “primary suspected” drug were identified within the reporting period. The search detected 276 disproportionate preferred terms (PTs). The most common AEs, including diarrhea, asthenia, decreased appetite, hypertension, and dysgeusia, were consistent with the drug label and clinical trials. Unexpected significant AEs, such as uveal melanocytic proliferation, salivary gland fistula, yellow skin, eyelash discoloration, scrotal inflammation, were detected. The median onset time of sunitinib-related AEs was 57 days (interquartile range [IQR]16–170 days), with most of the ICSRs developing within the first month (n = 4,582, 39.73%) after sunitinib therapy as initiated.

**Conclusion:**

The results of our study were consistent with routine clinical observations, and some unexpected AEs signals were also identified for sunitinib, providing valuable evidence for the safe use of sunitinib in the real-world and contributing to the clinical monitoring and risk identification of sunitinib.

## 1 Introduction

Sunitinib, an oral small-molecule multitargeted tyrosine kinase inhibitors (TKIs), exerts dual effects on anti-tumor angiogenesis and anti-tumor cell proliferation via inhibiting the vascular endothelial growth factor-1, 2, 3 (VEGF-1, 2, 3), the platelet-derived growth factor-α, β (PDGFr-α, β), the stem-cell growth factor receptor (KIT), colony-stimulating factor (CSF)-1R, FMS-like tyrosine kinase-3 (FLT-3), and the rearranged during transfection (RET) (Mena et al., 2010). The agent has been approved by the Food and Drug Administration (FDA) for the treatment of metastatic renal cell carcinoma (mRCC), gastrointestinal stromal tumor (GIST) refractory to imatinib therapy, and advanced pancreatic neuroendocrine tumor (PNET) (Raymond et al., 2011; Savard et al., 2020; Li et al., 2024). The available data suggests that it can significantly prolong the median progression-free survival and overall survival of patients with mRCC or GIST (Hopkins et al., 2008; Moran et al., 2019). Encouraging results have also been shown in clinical trials of sunitinib alone or in combination with other anti-tumour agents for the treatment of solid tumours such as lung and breast cancer (Crown et al., 2013; Tanday, 2015).

However, despite its significant clinical benefits, the widespread clinical use of sunitinib inevitably leads to adverse effects in patients. In clinical phase II and phase III studies of sunitinib, the most common adverse events (AEs) occurring in at least 25% of recipients include fatigue, rash, diarrhea, mucositis, loss of appetite, hand-foot syndrome, hypertension, hemorrhages, taste disturbances, and dyspepsia (Cella et al., 2008; Escudier et al., 2009). Additionally, sunitinib has an FDA “black box” warning for hepatotoxicity, which has the potential to be fatal (Amaya et al., 2018). Therefore, it is highly desirable to utilize data mining algorithms to identify potential safety signals of sunitinib in real-world settings.

The Food and Drug Administration Adverse Event Reporting System (FAERS) is one of the largest post-marketing safety monitoring databases that document real-world standardized data, which can be used for identifying and analyzing potential drug-AEs associations (Michel et al., 2017). We performed a retrospective pharmacovigilance study to detect the AEs signals of sunitinib based on disproportionality analysis methods. This research aimed to find unexpected AEs that were not described on the drug’s label and offer valuable reference for clinical practices.

## 2 Materials and methods

### 2.1 Data source and processing

**FIGURE 1:**
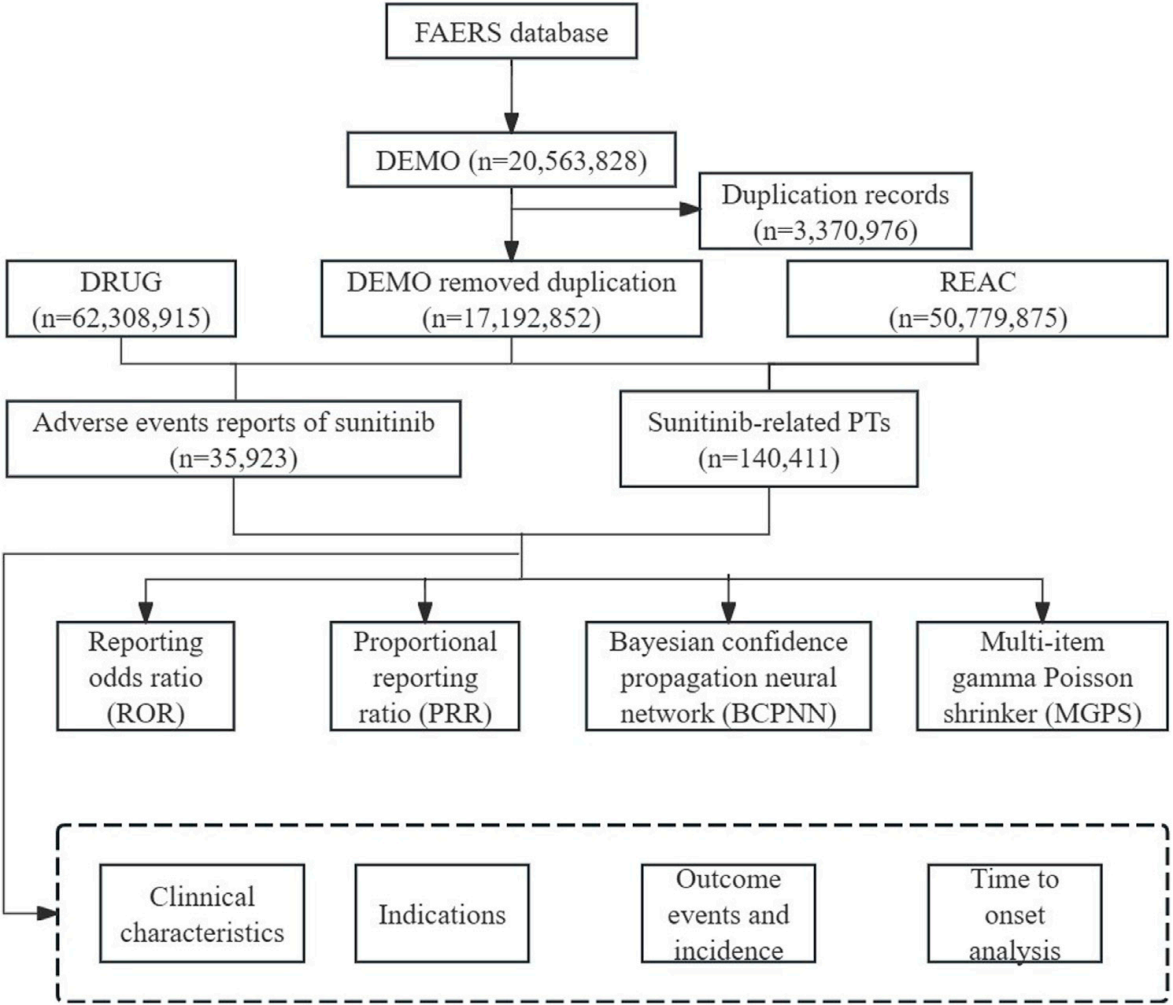
Flowchart of identifying AE cases of sunitinib from the FAERS database.

FAERS is the primary system in the United States for conducting post-marketing adverse drug reaction surveillance and is one of the main avenues for current pharmacovigilance research (Dhodapkar et al., 2022). In our pharmacovigilance study, the ASCII data packages submitted from the first quarter of 2006 to the first quarter of 2024 were retrieved from the database. All data analyses were imported into SAS 9.4 and Excel software for data cleaning and analyses. The sample group was chosen by screening for DRUGNAME and PROD_AI, using both the generic and brand names (sunitinib, Sutent^®^) as keywords, with the suspicion level for reporting limited to “primary suspect” drugs. Based on the latest version of the Medical Dictionary for Regulatory Activities (MedDRA) 26.0 dictionary, AEs of sunitinib are coded on preferred terms (PT) and system organ class (SOC) levels, and the toxicity spectrum of sunitinib was investigated. According to the recommended approach by the FDA to remove duplicate reports, choose the PRIMARYID, CASEID, and FDA_DT fields of the DEMO table, sort them by CASEID, FDA_DT, and PRIMARYID, and keep the report with the highest FDA_DT value for individual case safety reports (ICSRs) with the same CASEID. Additionally, keep the one with the highest PRIMARYID value for reports with both the same CASEID and FDA_DT. Ensure retention of the one with the largest PRIMARYID value. Since Q1 2019, a roster of erased reports has been included in every quarterly packet. Following data de-duplication, reports are deleted using the CASEID included in the roster above. The comprehensive screening procedure was depicted in Figure 1.

### 2.2 Onset time analysis

The onset time of sunitinib-related events was calculated by subtracting the date of AE occurrence from the date of sunitinib initiation. It is crucial to highlight that the study excluded erroneous ICSRs whose occurrence date preceded the initiation of the drug or ICSRs of unknown time of onset. The median and interquartile ranges were employed to characterize the time to onset.

### 2.3 Statistical analysis

Descriptive analyses were used to present all sunitinib-related AEs reporting characteristics. In our study, disproportionate analyses, including reporting odds ratio (ROR) (Sakaeda et al., 2013), proportional reporting ratio (PRR) (Kelly et al., 2007), bayesian confidence propagation neural network (BCPNN) (Bate et al., 1998), and multinomial gamma Poisson shrinkage (MGPS) (Szarfman et al., 2004), were performed to identify signals indicating a possible increased risk of sunitinib-related AEs. The ROR and PRR are classified as frequency methods, and the methods demonstrate high sensitivity but low specificity. The BCPNN and MGPS are categorized as Bayesian methods which are suitable for handling complex variables, but with low sensitivity (Zou et al., 2023). Accordingly, multiple algorithms are combined to ensure the stability and reliability of the research results. The larger the value of the four parameters, the stronger the signal strength is. The relevant numerical values are the signal strength. The formulas and criteria for the four algorithms are shown in Table 1. (Liu et al., 2024; Tang et al., 2024). The AEs uncovered in the latest version of the sunitinib label issued by the FDA were defined as unexpected AEs. (FDA LABEL, 2021).

**TABLE 1:** The specific formulas for the four algorithms.

Algorithms	Equation	Criteria
ROR	ROR = ad/bc	95%CI > 1, N ≥ 3
	95%CI = e^ln(ROR)±1.96(1/a+1/b+1/c+1/d) ^ 0.5^	
PRR	PRR = a (c + d)/c (a+b)	PRR≥2, χ^2^ ≥ 4, N ≥ 3
	χ^2^ = [(ad-bc)^^^2](a+b + c + d)/[(a+b) (c + d) (a+c) (b + d)]	
BCPNN	IC = log_2_a (a + b + c + d) (a + c) (a + b)	PRR≥2, χ^2^ ≥ 4, N ≥ 3, IC025 > 0
	95% CI = e^ln(IC)±1.96(1/a+1/b+1/c+1/d) ^ 0.5^	
MGPS	EBGM = a (a + b + c + d)/(a + c)/(a + b)	EBGM05 > 2, N > 0
	95% CI = e^ln(EBGM)±1.96(1/a+1/b+1/c+1/d) ^ 0.5^	

Equation: a, number of reports containing both the target drug and the target adverse drug reaction; b, number of reports containing other adverse drug reactions of the target drug; c, number of reports containing the target adverse drug reaction of other drugs; d, number of reports containing other drugs and other adverse drug reactions. The MGPS, employs an empirical Bayesian approach, whereby a prior distribution is obtained by maximum likelihood estimates, and the prior and likelihood are subsequently combined to obtain a posterior distribution. The fifth percentile of the posterior distribution is denoted by “EBGM05” and is interpreted as the one-sided 95%confidence lower bound for the EBGM.

Abbreviations: 95% CI, 95% confidence interval; N, the number of reports; χ^2^, chi-squared; IC, information component; IC025, the lower limit of the 95% CI, of the IC; E (IC), the IC, expectations; V (IC), the variance of IC; EBGM, empirical Bayesian geometric mean; EBGM05, empirical Bayesian geometric mean lower 95% CI, for the posterior distribution.

## 3 Results

### 3.1 Descriptive analysis

**FIGURE 2:**
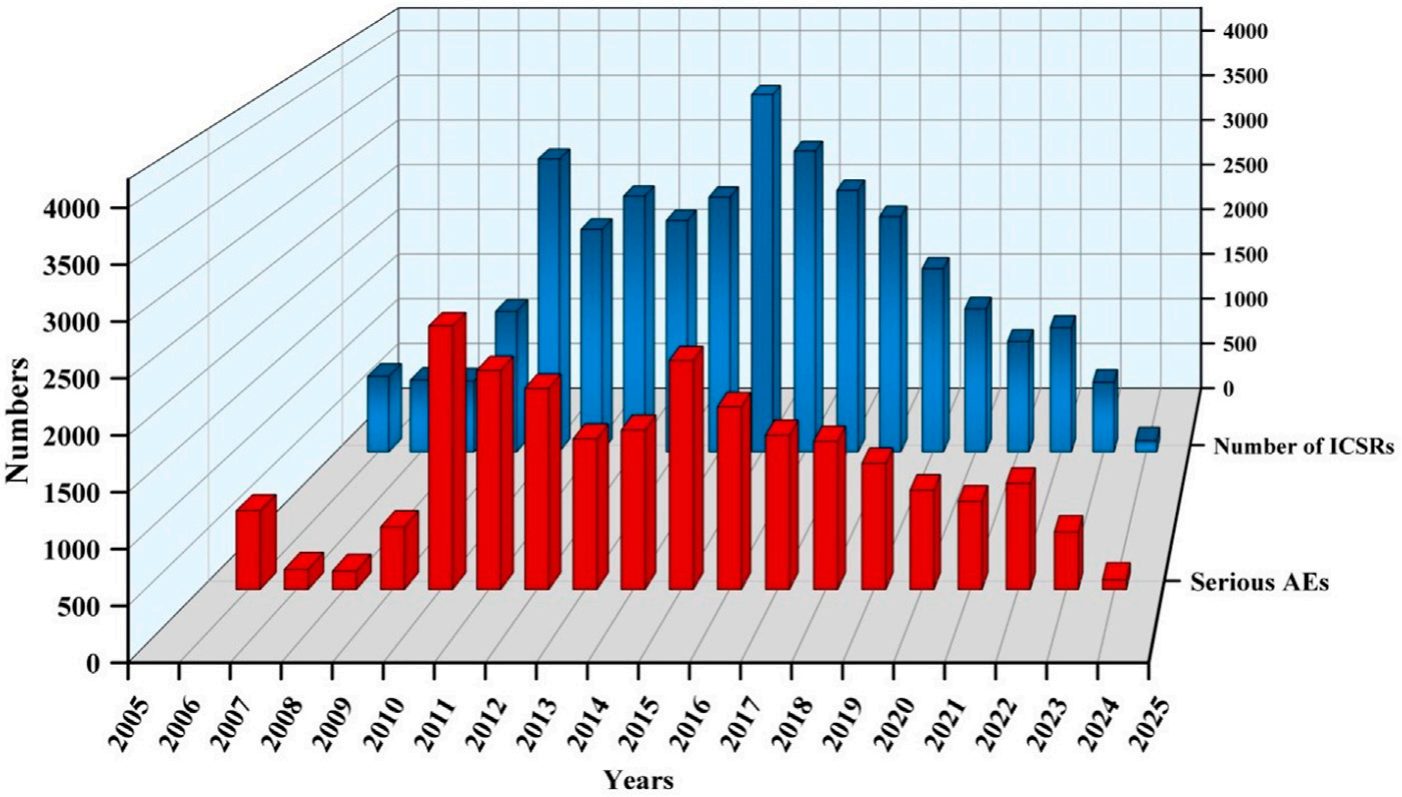
Annual trends in sunitinib reporting.

A total of 20,563,828 ICSRs were submitted to the FAERS database during the study period, among which there were 35,923 ICSRs on sunitinib of PS. The clinical characteristics of events regarding sunitinib were presented in Table 2 and Figure 2. Among all ICSRs, more males (59.35%) than females (31.85%) were reported. In terms of age, patients aged>65 contributed to the majority of ICSRs (38.55%). Death (28.92%) and hospitalization-initial or prolonged (28.38%) were the most common serious outcomes, and the high proportion of deaths might be more related to cancer progression in AEs. The country that reported the most was the United States (48.87%), and the primary ICSRs were consumers (38.13%) and physicians (30.93%). In terms of year of reporting, ICSRs were concentrated in 2010 (3085 cases), 2015 (3,760 cases), and 2016 (3,167 cases).

**TABLE 2:** Clinical characteristics of reports with sunitinib from the FAERS database.

Characteristics	ICSRs number, n	ICSRs proportion, %
Gender (n = 35,923)		
Male	21320	59.35
Female	11441	31.85
Unknown	3162	8.80
Age (n = 35,923)		
<18	59	0.16
18–44	1716	4.78
45–64	11636	32.39
≥65	13847	38.55
Unknown	8665	24.12
Serious outcomes (AEs case number, n = 34,973)		
Death	10392	28.92
Hospitalization-initial or prolonged	10283	28.38
Disability	360	1.00
Life-threatening	1080	3.01
Other serious	12858	35.79
Reported Countries (Top five, n = 24,641)		
United States	17555	48.87
Japan	1980	5.51
China	1854	5.16
Argentina	1833	5.10
India	1419	3.95
Reported Person (n = 35,923)		
Consumer	13696	38.13
Physician	11111	30.93
Other health-professional	6127	17.09
Pharmacist	3786	10.54
Lawyer	4	0.01
Missing	1199	3.34
Indications (TOP five, n = 22,582)		
Metastatic renal cell carcinoma	7361	20.49
Renal cell carcinoma	6709	19.07
Renal cancer	4,348	18.68
Gastrointestinal stromal tumour	2792	12.10
Renal cancer metastatic	1372	3.82

### 3.2 Potential safety signal detection results

We performed signal detection of sunitinib-associated AEs at the SOC level which was shown in Table 3. AEs of sunitinib involved a total of 27 organ systems. Statistically, the SOC that met all four criteria simultaneously and showed significant association with sunitinib AEs were gastrointestinal disorders (n = 24452, ROR 2.27, PRR 2.04, IC 1.03, EBGM 2.04) and endocrine disorders (n = 1128, ROR 3.21, PRR 3.21, IC 1.67, EBGM 3.18). Additionally, general disorders and administration site conditions (n = 28170) were the most common SOCs. Of note, skin and subcutaneous tissue disorders (n = 10290), blood and lymphatic system disorders (n = 4,123), and vascular disorders (n = 3864) were also common and noteworthy SOC categories.

**TABLE 3:** Signal strength of AEs of sunitinib at the SOC level in the FAERS database.

System organ class (SOC)	Case numbers (n = 140,411)	ROR (95% two-side Cl)	PRR (χ2)	IC (IC025)	EBGM (EBGM05)
General disorders and administration site conditions	28170	1.18 (1.16–1.19) *	1.14 (617.38)	0.19 (0.17) *	1.14 (1.12)
Gastrointestinal disorders	24452	2.27 (2.24–2.30) *	2.04 (1401.15) *	1.03 (1.01) *	2.04 (2.01)*
Investigations	11564	1.40 (1.37–1.42) *	1.36 (1189.21)	0.45 (0.42) *	1.36 (1.34)
Skin and subcutaneous tissue disorders	10290	1.39 (1.33–1.38) *	1.36 (1018.48)	0.44 (0.41) *	1.36 (1.33)
Nervous system disorders	9701	0.80 (0.78–0.82)	0.81 (450.90)	−0.29 (−0.33)	0.81 (0.79)
Neoplasms benign, malignant and unspecified (incl cysts and polyps)	7050	1.91 (1.87–1.96) *	1.87 (2892.42)	0.90 (0.86) *	1.86 (1.82)
Respiratory, thoracic and mediastinal disorders	6495	0.98 (0.96–1.01)	0.99 (1.47)	−0.02 (−0.06)	0.98 (0.96)
Metabolism and nutrition disorders	5564	1.87 (1.82–1.91) *	1.83 (2149.89)	0.87 (0.83)*	1.83 (1.78)
Musculoskeletal and connective tissue disorders	5116	0.69 (0.67–0.70)	0.70 (700.66)	−0.52 (−0.56)	0.70 (0.68)
Infections and infestations	4,760	0.63 (0.62–0.65)	0.65 (973.03)	−0.63 (−0.67)	0.65 (0.63)
Injury, poisoning and procedural complications	4,622	0.30 (0.29–0.31)	0.33 (7109.71)	−1.62 (−1.66)	0.33 (0.32)
Blood and lymphatic system disorders	4,123	1.79 (1.74–1.85) *	1.77 (13885.38)	0.82 (0.77) *	1.77 (1.71)
Vascular disorders	3864	1.29 (1.25–1.33) *	1.28 (238.31)	0.35 (0.31)*	1.28 (1.24)
Renal and urinary disorders	2794	1.03 (1.00–1.06) *	1.02 (1.32)	0.031 (−0.024)	1.02 (0.98)
Cardiac disorders	2742	0.73 (0.71–0.76)	0.74 (257.84)	−0.44 (−0.49)	0.74 (0.71)
Psychiatric disorders	2667	0.32 (0.31–0.34)	0.34 (3690.17)	−1.57 (−1.63)	0.34 (0.32)
Hepatobiliary disorders	1675	1.33 (1.26–1.40) *	1.33 (135.25)	0.41 (0.34) *	1.33 (1.26)
Eye disorders	1501	0.53 (0.51–0.56)	0.54 (595.38)	−0.89 (−0.96)	0.54 (0.51)
Endocrine disorders	1128	3.21 (3.02–3.41) *	3.21 (1690.39) *	1.67 (1.60) *	3.18 (2.99) *
Reproductive system and breast disorders	571	0.45 (0.42–0.49)	0.45 (393.66)	−1.16 (−1.28)	0.45 (0.41)
Surgical and medical procedures	497	0.26 (0.24–0.29)	0.27 (992.68)	−1.90 (−2.03)	0.27 (0.24)
Ear and labyrinth disorders	365	0.59 (0.54–0.66)	0.60 (99.72)	−0.75 (−0.90)	0.60 (0.54)
Immune system disorders	358	0.23 (0.21–0.26)	0.23 (910.69)	−2.10 (−2.24)	0.23 (0.21)
Social circumstances	235	0.36 (0.32–0.41)	0.37 (256.87)	−1.45 (−1.63)	0.37 (0.32)
Product issues	54	0.023 (0.017–0.031)	0.024 (2119.542)	−5.37 (−5.73)	0.024 (0.09)
Congenital, familial and genetic disorders	46	0.11 (0.080–0.14)	0.11 (346.16)	−3.23 (−3.62)	0.11 (0.080)
Pregnancy, puerperium and perinatal conditions	7	0.011 (0.0055–0.024)	0.012 (595.81)	−6.43 (−7.26)	0.012 (0.0055)

*Indicates statistically significant signals in the algorithm.

Based on the signal frequency and the signal strength (adopting the most sensitive ROR algorithm results), we respectively ranked these AEs which satisfied all four screening methods, as detailed in Tables 4, 5. Excepting in AEs of death (n = 6873), in our results, diarrhea (n = 4,049), fatigue (n = 3793), asthenia (n = 2249), decreased appetite (n = 2222), hypertension (n = 1678), and dysgeusia (n = 1606) were the most common AEs, which were consistent with the label and clinical trials. The AEs with significant potential risk signals included diffuse uveal melanocytic proliferation (ROR = 131.15, PRR = 131.15, IC = 6.59, EBGM = 96.44), salivary gland fistula (ROR = 98.36, PRR = 98.36, IC = 6.28, EBGM = 77.50), yellow skin (ROR = 67.81, PRR = 67.50, IC = 5.83, EBGM = 57.02), eyelash discoloration (ROR = 64.03, PRR = 64.02, IC = 5.77, EBGM = 54.52), scrotal inflammation (ROR = 39.20, PRR = 39.20, IC = 5.15, EBGM = 35.46), among others. Furthermore, comparing with the latest package insert of released by the FDA, unexpected significant AEs were discovered, such as including diffuse uveal melanocytic proliferation (ROR = 131.15, PRR = 131.15, IC = 6.59, EBGM = 96.44), salivary gland fistula (ROR = 98.36, PRR = 98.36, IC = 6.28, EBGM = 77.50), scrotal inflammation (ROR = 39.20, PRR = 39.20, IC = 5.15, EBGM = 35.46), thyroid atrophy (ROR = 36.07, PRR = 36.07, IC = 5.04, EBGM = 32.88), esophagobronchial fistula (ROR = 36.07, PRR = 36.07, IC = 5.04, EBGM = 32.88), perihepatic discomfort (ROR = 15.03, PRR = 15.03, IC = 3.85, EBGM = 14.47) and vena cava injury (ROR = 14.24, PRR = 14.24, IC = 3.78, EBGM = 13.73). Of note, nausea, vomiting, malaise, pain in extremity, pyrexia, constipation, and abdominal pain in the drug label did not meet the criteria for at least one of the four algorithms.

**TABLE 4:** The top 50 AEs of sunitinib ranked by the frequency at the PTs level.

SOC	PTs	Case numbers (n = 45,773)	ROR (95% two-sided Cl)	PRR (χ2)	IC (IC025)	EBGM (EBGM05)
General disorders and administration site conditions	Death	6873	3.62 (2.11–3.71)	3.5 (12299.04)	1.80 (1.76)	3.47 (3.39)
Gastrointestinal disorders	Diarrhoea	4,049	2.87 (2.78–2.96)	2.82 (4,752.36)	1.49 (1.44)	2.80 (2.71)
General disorders and administration site conditions	Fatigue	3793	2.18 (2.11–2.25)	2.15 (2347.29)	1.10 (1.05)	2.14 (2.07)
General disorders and administration site conditions	Asthenia	2249	2.67 (2.56–2.78)	2.64 (2296.21)	1.40 (1.33)	2.63 (2.52)
Metabolism and nutrition disorders	Decreased appetite	2222	4.12 (3.95–4.3)	4.07 (5116.74)	2.01 (1.95)	4.04 (3.87)
Vascular disorders	Hypertension	1678	3.56 (3.39–3.73)	3.53 (3016.82)	1.81 (1.73)	3.50 (3.34)
Nervous system disorders	Dysgeusia	1606	9.3 (8.85–9.77)	9.2 (11467.07)	3.17 (3.09)	9.00 (8.56)
Investigations	Weight decreased	1409	2.21 (2.1–2.33)	2.2 (920.3)	1.13 (1.05)	2.19 (2.08)
Skin and subcutaneous tissue disorders	Palmar-plantar erythrodysaesthesia syndrome	1378	26.88 (25.44–28.4)	26.63 (31664.21)	4.64 (4.53)	24.87 (23.54)
Gastrointestinal disorders	Stomatitis	1350	10.29 (9.75–10.86)	10.2 (10905.41)	3.31 (3.22)	9.95 (9.42)
Investigations	Platelet count decreased	1245	5.23 (4.95–5.54)	5.2 (4,165.12)	2.36 (2.27)	5.14 (4.86)
Investigations	Blood pressure increased	1130	3.26 (3.07–3.45)	3.24 (1736.02)	1.69 (1.60)	3.22 (3.03)
Gastrointestinal disorders	Oral pain	1089	21.71 (20.41–23.08)	21.55 (20140.34)	4.35 (4.23)	20.39 (19.17)
Metabolism and nutrition disorders	Dehydration	1002	3.31 (3.11–3.52)	3.29 (1585.43)	1.71 (1.61)	3.27 (3.07)
Blood and lymphatic system disorders	Thrombocytopenia	955	3.91 (3.66–4.16)	3.89 (2028.63)	1.95 (1.85)	3.86 (3.62)
Gastrointestinal disorders	Dyspepsia	781	3.59 (3.35–3.86)	3.58 (1439.75)	1.83 (1.72)	3.55 (3.31)
Investigations	White blood cell count decreased	719	2.93 (2.72–3.15)	2.92 (902.18)	1.54 (1.43)	2.90 (2.7)
Skin and subcutaneous tissue disorders	Dry skin	706	2.47 (2.29–2.66)	2.46 (608.89)	1.29 (1.18)	2.45 (2.27)
Skin and subcutaneous tissue disorders	Blister	692	5.69 (5.28–6.14)	5.67 (2621.03)	2.48 (2.36)	5.60 (5.19)
Respiratory, thoracic and mediastinal disorders	Epistaxis	674	3.92 (3.63–4.23)	3.91 (1443.35)	1.95 (1.84)	3.87 (3.59)
Skin and subcutaneous tissue disorders	Yellow skin	650	67.81 (62.35–73.74)	67.5 (35873.37)	5.83 (5.59)	57.02 (52.43)
Endocrine disorders	Hypothyroidism	606	8.83 (8.15–9.57)	8.8 (4,090.25)	3.11 (2.97)	8.61 (7.94)
Nervous system disorders	Ageusia	596	10.58 (9.75–11.48)	10.54 (5001.91)	3.36 (3.22)	10.27 (9.46)
Respiratory, thoracic and mediastinal disorders	Pleural effusion	585	4.23 (3.9–4.59)	4.22 (1419.72)	2.06 (1.93)	4.18 (3.85)
Skin and subcutaneous tissue disorders	Skin exfoliation	581	3.16 (2.91–3.43)	3.15 (846.26)	1.65 (1.52)	3.13 (2.89)
Gastrointestinal disorders	Glossodynia	572	13.51 (12.42–14.68)	13.45 (6359.14)	3.7 (3.55)	13.01 (11.96)
General disorders and administration site conditions	Mucosal inflammation	457	8 (7.29–8.78)	7.98 (2729.34)	2.97 (2.81)	7.83 (7.13)
Gastrointestinal disorders	Dry mouth	452	2.5 (2.28–2.75)	2.5 (403.84)	1.31 (1.17)	2.49 (2.27)
Skin and subcutaneous tissue disorders	Hair colour changes	436	14.22 (12.92–15.65)	14.18 (5140.93)	3.77 (3.59)	13.68 (12.43)
Investigations	Blood creatinine increased	419	2.84 (2.58–3.13)	2.84 (495.57)	1.50 (1.35)	2.82 (2.56)
Skin and subcutaneous tissue disorders	Skin discolouration	412	3.91 (3.55–4.31)	3.9 (881.1)	1.95 (1.8)	3.87 (3.51)
Gastrointestinal disorders	Ascites*	372	5.64 (5.09–6.25)	5.63 (1394.16)	2.47 (2.31)	5.56 (5.01)
Gastrointestinal disorders	Flatulence	341	2.67 (2.4–2.97)	2.66 (352.26)	1.41 (1.24)	2.65 (2.38)
Blood and lymphatic system disorders	Bone marrow failure*	267	5.2 (4.61–5.87)	5.19 (891.59)	2.36 (2.16)	5.13 (4.55)
General disorders and administration site conditions	Multiple organ dysfunction syndrome	261	2.61 (2.31–2.94)	2.6 (255.85)	1.37 (1.19)	2.59 (2.29)
Gastrointestinal disorders	Mouth ulceration	255	5.52 (4.88–6.25)	5.52 (928.89)	2.45 (2.24)	5.45 (4.81)
Hepatobiliary disorders	Jaundice	254	4.15 (3.67–4.7)	4.15 (600.03)	2.04 (1.84)	4.11 (3.63)
Gastrointestinal disorders	Oral discomfort	251	7.85 (6.92–8.89)	7.83 (1465.1)	2.94 (2.72)	7.69 (6.78)
Psychiatric disorders	Eating disorder	239	4.83 (4.25–5.49)	4.83 (715.84)	2.26 (2.05)	4.78 (4.2)
Skin and subcutaneous tissue disorders	Hyperkeratosis	223	18.24 (15.95–20.87)	18.22 (3454.35)	4.12 (3.82)	17.39 (15.2)
Renal and urinary disorders	Chromaturia	211	3.97 (3.47–4.55)	3.97 (463.37)	1.98 (1.76)	3.94 (3.44)
Endocrine disorders	Thyroid disorder	208	5.98 (5.22–6.86)	5.97 (847.68)	2.56 (2.32)	5.89 (5.14)
Skin and subcutaneous tissue disorders	Skin lesion	205	3.35 (2.92–3.84)	3.34 (333.97)	1.73 (1.51)	3.32 (2.9)
Skin and subcutaneous tissue disorders	Skin disorder	203	2.71 (2.36–3.11)	2.71 (217.25)	1.43 (1.22)	2.70 (2.35)
Renal and urinary disorders	Haematuria	196	2.44 (2.12–2.81)	2.44 (165.37)	1.28 (1.06)	2.43 (2.11)
General disorders and administration site conditions	Impaired healing	192	2.72 (2.36–3.13)	2.71 (206.3)	1.43 (1.21)	2.70 (2.34)
Metabolism and nutrition disorders	Feeding disorder	186	4.61 (3.99–5.33)	4.61 (518.95)	2.19 (1.95)	4.56 (3.95)
Investigations	Red blood cell count decreased	183	2.81 (2.43–3.25)	2.8 (210.83)	1.48 (1.25)	2.79 (2.41)
Respiratory, thoracic and mediastinal disorders	Haemoptysis	181	2.81 (2.42–3.25)	2.8 (208.41)	1.48 (1.25)	2.79 (2.41)
Skin and subcutaneous tissue disorders	Skin fissures	179	4.89 (4.22–5.67)	4.89 (545.89)	2.27 (2.03)	4.83 (4.17)

*, AEs, that are not mentioned in the drug label.

**TABLE 5:** The top 50 signal strength of AEs of sunitinib ranked by the ROR at the PTs level.

SOC	PTs	Case numbers (n = 8,920)	ROR (95% two-sided Cl)	PRR (χ2)	IC (IC025)	EBGM (EBGM05)
Eye disorders	Diffuse uveal melanocytic proliferation*	4	131.15 (41.76–411.89)	131.15 (378.85)	6.59 (0.78)	96.44 (30.71)
Gastrointestinal disorders	Salivary gland fistula*	3	98.36 (27.44–352.58)	98.36 (227.16)	6.28 (0.32)	77.50 (21.62)
Skin and subcutaneous tissue disorders	Yellow skin	650	67.81 (62.35–73.74)	67.5 (35873.37)	5.83 (5.59)	57.02 (52.43)
Eye disorders	Eyelash discolouration	30	64.03 (43.43–94.42)	64.02 (1580.54)	5.77 (3.76)	54.52 (36.97)
Reproductive system and breast disorders	Scrotal inflammation*	5	39.20 (15.58–98.66)	39.2 (167.89)	5.15 (1.15)	35.46 (14.09)
Injury, poisoning and procedural complications	Anal injury	14	39.14 (22.55–67.95)	39.14 (469.39)	5.15 (2.64)	35.41 (20.4)
Endocrine disorders	Thyroid atrophy*	5	36.07 (14.38–90.44)	36.07 (154.97)	5.04 (1.14)	32.88 (13.11)
Skin and subcutaneous tissue disorders	Plantar erythema	18	32.14 (19.85–52.05)	32.14 (498.6)	4.89 (2.87)	29.59 (18.27)
Reproductive system and breast disorders	Penile exfoliation	5	30.56 (12.27–76.16)	30.56 (131.81)	4.82 (1.12)	28.25 (11.34)
Skin and subcutaneous tissue disorders	Palmar-plantar erythrodysaesthesia syndrome	1378	26.88 (25.44–28.4)	26.63 (31664.21)	4.64 (4.53)	24.87 (23.54)
Endocrine disorders	Myxoedema	11	24.64 (13.38–45.39)	24.64 (233.54)	4.53 (2.16)	23.13 (12.56)
Injury, poisoning and procedural complications	Mouth injury	78	23.65 (18.81–29.74)	23.64 (1587.18)	4.48 (3.8)	22.25 (17.69)
Gastrointestinal disorders	Oral pain	1089	21.71 (20.41–23.08)	21.55 (20140.34)	4.35 (4.23)	20.39 (19.17)
Cardiac disorders	Cardiopulmonary failure	164	18.54 (15.85–21.7)	18.52 (2586.13)	4.14 (3.77)	17.67 (15.1)
Skin and subcutaneous tissue disorders	Hyperkeratosis*	223	18.24 (15.95–20.87)	18.22 (3454.35)	4.12 (3.82)	17.39 (15.2)
Musculoskeletal and connective tissue disorders	Jaw fistula	5	16.7 (6.81–40.93)	16.70 (70.52)	4 (0.98)	16 (6.53)
Reproductive system and breast disorders	Scrotal erythema	6	16.39 (7.23–37.15)	16.39 (82.96)	3.97 (1.22)	15.72 (6.94)
Skin and subcutaneous tissue disorders	Splinter haemorrhages*	5	16.10 (6.57–39.44)	16.1 (67.79)	3.95 (0.97)	15.46 (6.31)
Hepatobiliary disorders	Perihepatic discomfort*	3	15.03 (4.73–47.7)	15.03 (37.71)	3.85 (0.24)	14.47 (4.56)
Reproductive system and breast disorders	Scrotal ulcer*	9	14.49 (7.44–28.22)	14.49 (108.67)	3.8 (1.67)	13.97 (7.17)
Reproductive system and breast disorders	Genital erythema	14	14.43 (8.46–24.62)	14.43 (168.21)	3.8 (2.14)	13.91 (8.15)
Injury, poisoning and procedural complications	Vena cava injury*	3	14.24 (4.49–45.13)	14.24 (35.52)	3.78 (0.23)	13.73 (4.33)
Skin and subcutaneous tissue disorders	Hair colour changes	436	14.22 (12.92–15.65)	14.18 (5140.93)	3.77 (3.59)	13.68 (12.43)
Gastrointestinal disorders	Anal ulcer	24	13.79 (9.17–20.72)	13.78 (274.06)	3.73 (2.57)	13.31 (8.86)
Gastrointestinal disorders	Glossodynia	572	13.51 (12.42–14.68)	13.45 (6359.14)	3.70 (3.55)	13.01 (11.96)
Nervous system disorders	Hypogeusia	60	13.35 (10.32–17.28)	13.35 (661)	3.69 (3.06)	12.91 (9.98)
Injury, poisoning and procedural complications	Genital injury	4	13.11 (4.84–35.57)	13.11 (43.19)	3.67 (0.61)	12.69 (4.68)
Respiratory, thoracic and mediastinal disorders	Mediastinal haemorrhage	3	13.04 (4.12–41.25)	13.04 (32.17)	3.66 (0.21)	12.62 (3.99)
Investigations	Haemoglobin	6	12.96 (5.74–29.26)	12.96 (63.91)	3.65 (1.13)	12.54 (5.56)
Respiratory, thoracic and mediastinal disorders	Hydrothorax	25	12.96 (8.69–19.31)	12.95 (266.23)	3.65 (2.54)	12.54 (8.41)
Skin and subcutaneous tissue disorders	Sweat discolouration	5	12.79 (5.24–31.21)	12.79 (52.48)	3.63 (0.89)	12.39 (5.08)
Gastrointestinal disorders	Rectal lesion	4	12.44 (4.59–33.7)	12.44 (40.66)	3.59 (0.59)	12.06 (4.45)
Gastrointestinal disorders	Tongue exfoliation	10	12.10 (6.45–22.73)	12.1 (98.54)	3.55 (1.68)	11.74 (6.25)
Gastrointestinal disorders	Oral mucosal roughening	6	12.02 (5.33–27.12)	12.02 (58.67)	3.54 (1.1)	11.67 (5.17)
Respiratory, thoracic and mediastinal disorders	Oesophagobronchial fistula*	4	12.02 (4.44–32.56)	12.02 (39.12)	3.54 (0.58)	11.67 (4.31)
Endocrine disorders	Primary hypothyroidism	5	11.79 (4.84–28.72)	11.79 (47.79)	3.52 (0.86)	11.44 (4.7)
Skin and subcutaneous tissue disorders	Umbilical haemorrhage*	4	11.73 (4.33–31.75)	11.73 (38.02)	3.51 (0.57)	11.39 (4.21)
Musculoskeletal and connective tissue disorders	Osteonecrosis of external auditory canal*	4	11.36 (4.2–30.73)	11.36 (36.63)	3.47 (0.56)	11.04 (4.08)
Gastrointestinal disorders	Gingival discomfort	15	11.27 (6.74–18.84)	11.27 (136.13)	3.45 (2.02)	10.96 (6.56)
Gastrointestinal disorders	Tongue blistering	57	10.75 (8.26–13.99)	10.75 (489.27)	3.39 (2.78)	10.46 (8.04)
Reproductive system and breast disorders	Genital lesion	10	10.70 (5.71–20.07)	10.7 (85.42)	3.38 (1.6)	10.42 (5.56)
Gastrointestinal disorders	Tongue discomfort	98	10.62 (8.68–12.98)	10.61 (828.75)	3.37 (2.94)	10.34 (8.45)
Nervous system disorders	Ageusia	596	10.58 (9.75–11.48)	10.54 (5001.91)	3.36 (3.22)	10.27 (9.46)
Gastrointestinal disorders	Anal inflammation	15	10.49 (6.27–17.52)	10.48 (125.06)	3.35 (1.96)	10.22 (6.11)
Investigations	Thyroid function test abnormal	105	10.42 (8.59–12.66)	10.42 (868.91)	3.34 (2.94)	10.15 (8.36)
Gastrointestinal disorders	Oral mucosal blistering	150	10.38 (8.83–12.21)	10.37 (1235.09)	3.34 (3.01)	10.11 (8.6)
Gastrointestinal disorders	Stomatitis	1350	10.29 (9.75–10.86)	10.2 (10905.41)	3.31 (3.22)	9.95 (9.42)
Investigations	Thyroglobulin increased	4	9.95 (3.68–26.87)	9.95 (31.33)	3.28 (0.51)	9.71 (3.6)
Injury, poisoning and procedural complications	Tongue injury	20	9.55 (6.13–14.9)	9.55 (149.22)	3.22 (2.1)	9.33 (5.99)
Nervous system disorders	Dysgeusia	1606	9.30 (8.85–9.77)	9.20 (11467.07)	3.17 (3.09)	9.00 (8.56)

*, AEs, that are not mentioned in the drug label.

### 3.3 Time-to-onset analysis

**FIGURE 3:**
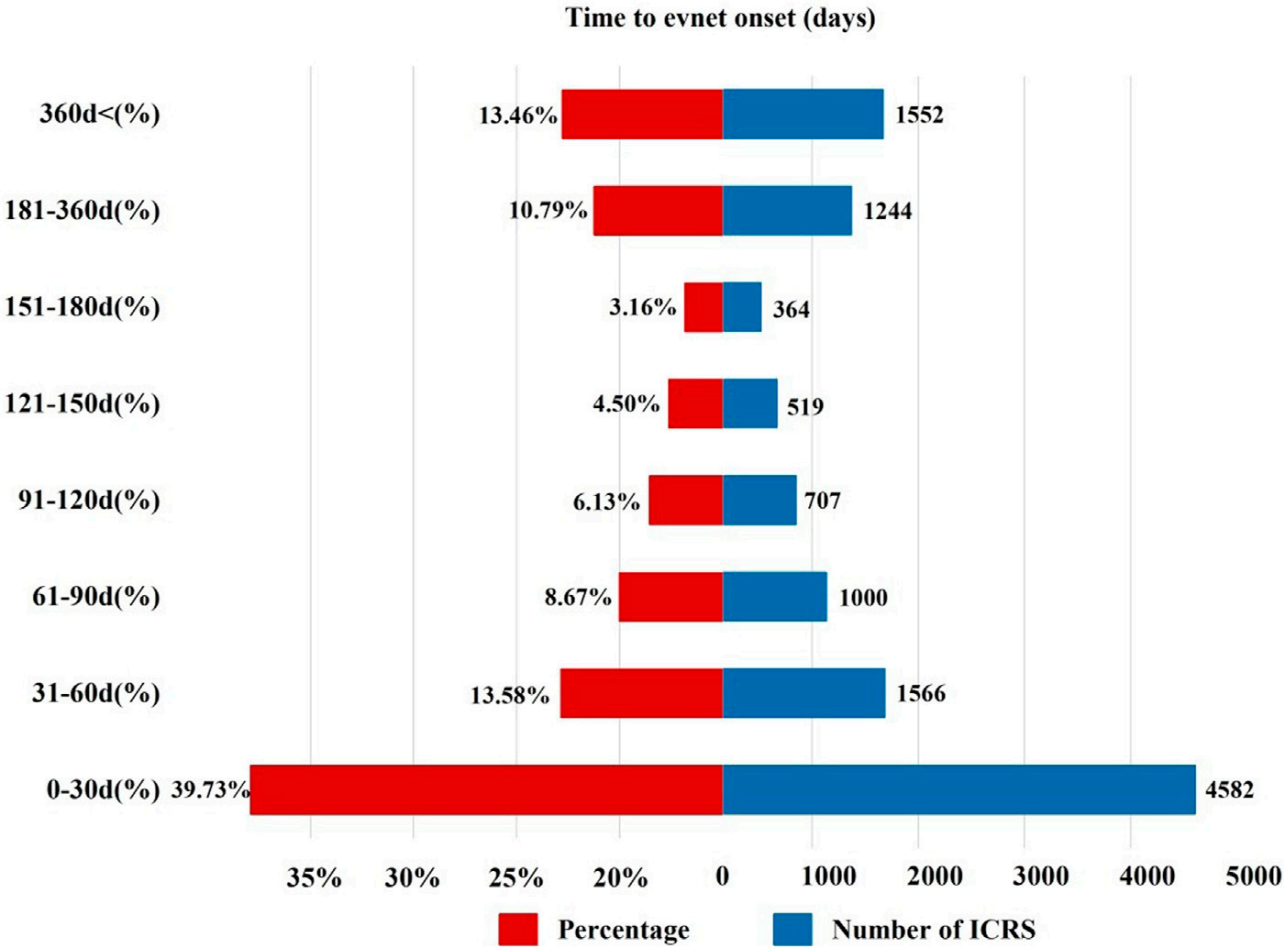
Time to onset of sunitinib-related AEs.

The onset time of sunitinib-related events was collected, with unreported onset time reports or erroneous reports excluded from the analysis. A total of 11,534 ICSRs were eligible for the inclusion criteria, and the mean time to onset was 207 days, with a median onset time of 51 days (interquartile range [IQR] 16–170 days). Our data revealed that 39.73% of ICSRs occurred within the first month following sunitinib administration (n = 4,582). Notably, AEs might still occur after 1 year of sunitinib treatment, accounting for 13.39% of the total cases, as illustrated in Figure 3.

## 4 Discussion

In recent years, several studies have carried out pharmacovigilance analyses of TKIs on electrolyte abnormalities, psychiatric disorders, and neuropsychiatric events (Raschi et al., 2022; Barbieri et al., 2023; Wang et al., 2023; She et al., 2024). However, the comprehensive safety profiles of sunitinib-induced AEs have not been reported. In this study, we systematically collect and assess the post-marketing AEs of sunitinib based on real-world pharmacovigilance data. The study aimed to identify new and significant risk signals and enhance the safety of clinical drug therapy. The ICSRs of sunitinib occurred more commonly in males (59.53%) than in females (31.85%), and a higher ICSRs proportion in elderly individuals over the age of 65 (38.83%), which could be attributed to the higher incidence of RCC in elderly males. It has been reported that the prevalence of renal cell carcinoma in males is approximately twice that in females and tends to increase with age (Scelo et al., 2018; Scelo and Larose, 2018). Besides, the median age of most patients at diagnosis is around 75 years old (Bukavina et al., 2022). Regarding reporting countries, the United States stands out as the most prominent reporting country (49.16%), possibly due to the earlier availability and higher prescription volume of the drug in the country. Notably, consumers (38.20%) were the predominant group reporting ICSRs, followed by physicians (30.98%). This indicates the need for heightening vigilance among clinical doctors and pharmacists in monitoring sunitinib-related AEs, particularly in the elderly population, to reduce the occurrence of life-threatening AEs.

Our disproportionality analyses identified that the significant SOCs were gastrointestinal disorders and endocrine disorders, and the most common SOC was general disorders and administration site conditions. As shown in Table 4, common AEs included diarrhea, fatigue, asthenia, decreased appetite, hypertension, dysgeusia, hand-foot syndrome, and stomatitis, which were mostly consistent with the insert and clinical trials (Saltz et al., 2007; Baumann et al., 2012; Jonasch et al., 2018). Hypertension is most common upon treatment with sunitinib (Pal et al., 2021). All-grade hypertension has been documented in up to 30% of mRCC patients receiving sunitinib, with grade 3 hypertension in 12% of cases (Motzer et al., 2009). Besides, hypertension was also a relatively common AE among other vascular endothelial growth factor TKIs (Zhu et al., 2009; Bæk Møller et al., 2019). The underlying mechanism is currently believed to be the activation of the endothelin-1 pathway and the disruption of endothelial cell survival signaling, resulting in reduced capillary density and diminished nitric oxide secretion (Kappers et al., 2010; Rini et al., 2011). It has been reported that hypertension often occurs as a complication in the early stages of treatment with TKIs, such as sorafenib and axitinib (Motzer et al., 2009). Therefore, it is recommended that blood pressure is regularly monitored at least once a week during the initial 6 weeks of sunitinib treatment (Zhu et al., 2009). In case of severe or persistent hypertension, it is advisable to withdraw sunitinib immediately and initiate antihypertensive treatment.

Some AEs with high signal intensity were also examined, such as yellow skin, eyelash discoloration, hand-foot syndrome (HFS), genital injury (including anal injuries, genital rashes, desquamation, ulcers, etc.), and bleeding events. Approximately 24% of patients suffered from skin discoloration, including yellow skin and pigmentation after sunitinib therapy (Rosenbaum et al., 2008). Of note, the yellow color of the medication may be a contributing factor to the development of yellow skin. Hair depigmentation occurred in 15.2% of patients, and the inhibition of multiple signaling pathways, such as platelet-derived growth factor receptors (PDGFR), seemed to have a role in the pathogenesis (Joensuu et al., 2011). The incidence of HFS was reported between 13.5% and 25%, of which 4%–7% of cases were grade 3 to 4. The onset of HFS was 2–4 weeks. The potential mechanism behind HFS is not completely understood, but it is hypothesized to involve the inhibition of VEGFR and PDGFR, resulting in vascular deformation and cell apoptosis in the dermis (Terada et al., 2015). Billemont et al. reported that 12.5% of patients experienced genital rash and anal injuries, with a median onset time of 66 days (Billemont et al., 2008). The mechanism of this AE is still largely unclear, and it may be related to VEGF and hypoxia-inducible factor-1a (Billemont et al., 2008; Chou et al., 2013). Lastly, various bleeding events such as splinter haemorrhage, umbilical haemorrhage, mediastinal haemorrhage, and scleral haemorrhage were also detected in our study. In a meta-analysis of patients with sunitinib and sorafenib, the incidence was 16.7% for all-grade bleeding events and 2.4% for grade 3/4 toxicity (Je et al., 2009). The increased risk of bleeding may be mediated by VEGF inhibition and concomitant administration with antiplatelet agents (Je et al., 2009). In addition, sunitinib-associated thrombocytopenia, with an incidence of 22% (Lee et al., 2014), was hypothesized to be an intriguing explanation for bleeding events.

Furthermore, unexpected and significant safety signals, such as diffuse uveal melanocytic proliferation, vena cava injury, esophagobronchial fistula, perihepatic discomfort, and thyroid atrophy, were detected in our analysis. Regardless, medical staff should note that patients on sunitinib are at risk of these unexpected AEs. So far, there have been no documented reports about diffuse uveal melanocytic proliferation and vena cava injury Therefore, it is necessary to further investigate the pathogenesis of these incidents. Basille et al. described a 40-year-old male with renal cell carcinoma who developed an esophagotracheal fistula after the administration of sunitinib for 2 months (Basille et al., 2010). However, the exact mechanism of esophagotracheal fistula remained unclear. In our study, we observed that sunitinib-induced hepatoxicity AEs mainly included perihepatic discomfort, liver failure, and jaundice. Approximately 40% of patients with sunitinib experienced elevated liver enzymes, and 3% of patients developed grade 3 or 4 hepatotoxicity (Ibrahim et al., 2013). Additionally, similar AEs have also been observed in other VEGF-TKIs (Teo et al., 2013). The onset time is 1–3 weeks after initiation of treatment or even several months later (Lammert et al., 2008). Toxic intermediate metabolites, mitochondrial dysfunction, and glycolysis inhibition have been described as possible mechanisms (Teo et al., 2013; Paech et al., 2017). Furthermore, several cases of sunitinib-related fulminant acute liver failure have been reported (Aqsa et al., 2021; Casas Deza et al., 2021). In 2010, FDA issued a black box warning that sunitinib-associated hepatotoxicity may be severe, and even fatal. Clinicians should be aware that liver failure is a complication unrelated to dosage or tumor progression, and liver function should be regularly monitored during the first year of treatment. According to a prospective study, the incidence of hypothyroidism as a common complication following sunitinib treatment was found to be 36% (Desai et al., 2006). However, our research findings suggested that sunitinib-induced thyroid atrophy represented a novel, unexpected, and rarely reported thyroid toxicity. A long-term study has indicated that the thyroid volume decreased by approximately 30% in metastatic renal cell carcinoma patients treated with sunitinib, and those with a reduction of over 50% in thyroid volume experienced a dramatically increased incidence of hypothyroidism (Shinohara et al., 2011). Thyroid atrophy induced by sunitinib may be attributed to the induction of follicular atrophy and apoptosis of thyroid cells, with its toxicity being a temporal relationship (Sakurai et al., 2010; Grosse et al., 2014). It has been observed that patients with sunitinib-induced hypothyroidism may show improvement upon suspension of the medication and initiation of levothyroxine therapy. However, in cases where severe thyroid atrophy caused by sunitinib leads to secondary hypothyroidism, it may be deemed irreversible (Desai et al., 2006). Therefore, regular monitoring of thyroid toxicity, particularly thyroid volume, is crucial during sunitinib treatment.

In this study, there is a wide variance between the onset of AEs and sunitinib application. The median onset time of reported AEs was 51 days, and 39.73% of ICSRs occurred within the first month after sunitinib initiation. However, 13.39% of ICSRs developed with a delayed onset, occurring 1 year after sunitinib therapy. Consistently, in a phase III clinical trial involving 312 GIST patients, the median onset time of AEs caused by sunitinib was 56 days, and the onset time of some AEs was longer. For example, the average onset time of hypothyroidism was 350 days (Joensuu et al., 2011). Therefore, it is imperative to be vigilant about the AEs throughout the whole course of treatment with sunitinib, and long-term follow-up for some AEs may be needed.

The pre-marketing drug safety studies are characterized by the small number of enrolled cases, brief observation periods, stringent medication usage conditions, *etc.* Thus, the AEs observed in pre-marketing trials of a drug may not reflect all AEs observed in practice. The spontaneous reporting database, with a wide monitoring range and early detection of suspected AE signals, was extensively used to carry out post-marketing pharmacovigilance on drug safety. In recent years, with the wide use of sunitinib in clinical practice there is an emerging need to make further evaluation of its safety profiles in a real-world environment. Our study first comprehensively and scientifically explores the post-marketing safety profiles of sunitinib based on AE reports from the FAERS database. Furthermore, some unexpected AEs were detected and the onset time of all AEs was analyzed, which guides the safe clinical use of sunitinib.

## 5 Conclusion

In conclusion, our study conducted a systematic and comprehensive exploration of the signals associated with sunitinib based on the FAERS database. The common AEs detected in this study were consistent with the manufacturer’s labeling and clinic trials. Some unexpected AEs were also revealed, such as diffuse uveal melanocytic proliferation, thyroid atrophy, esophagobronchial fistula, vena cava injury, and perihepatic discomfort. Furthermore, the median onset time of AEs was analyzed, which enables clinicians and pharmacists to make informed decisions regarding sunitinib. However, given the exploratory character of our work, future prospective clinical trials and long-term data are needed to validate these results and provide valuable evidence for the safety profile of sunitinib.

## Data Availability

The original contributions presented in the study are included in the article/Supplementary Material, further inquiries can be directed to the corresponding authors.
